# Patterns of Gene Content and Co-occurrence Constrain the Evolutionary Path toward Animal Association in Candidate Phyla Radiation Bacteria

**DOI:** 10.1128/mBio.00521-21

**Published:** 2021-07-13

**Authors:** Alexander L. Jaffe, Alex D. Thomas, Christine He, Ray Keren, Luis E. Valentin-Alvarado, Patrick Munk, Keith Bouma-Gregson, Ibrahim F. Farag, Yuki Amano, Rohan Sachdeva, Patrick T. West, Jillian F. Banfield

**Affiliations:** a Department of Plant and Microbial Biology, University of California, Berkeley, Berkeley, California, USA; b Department of Environmental Science, Policy, and Management, University of California, Berkeley, Berkeley, California, USA; c Rocky Mountain Biological Laboratory, Crested Butte, Colorado, USA; d Innovative Genomics Institute, University of California, Berkeley, Berkeley, California, USA; e Department of Civil and Environmental Engineering, University of California, Berkeley, Berkeley, California, USA; f National Food Institute, Technical University of Denmark, Kongens Lyngby, Denmark; g Department of Earth and Planetary Science, University of California, Berkeley, Berkeley, California, USA; h Department of Integrative Biology, University of California, Berkeley, Berkeley, California, USA; i School of Marine Science and Policy, University of Delaware, Lewes, Delaware, USA; j Nuclear Fuel Cycle Engineering Laboratories, Japan Atomic Energy Agency, Ibaraki, Japan; k Horonobe Underground Research Center, Japan Atomic Energy Agency, Hokkaido, Japan; l Department of Medicine (Hematology & Blood and Marrow Transplantation), Stanford University, Stanford, California, USA; m Chan Zuckerberg Biohub, San Francisco, California, USA; Oregon State University

**Keywords:** CPR bacteria, animal microbiome, bacterial evolution, comparative genomics, habitat transition

## Abstract

Candidate Phyla Radiation (CPR) bacteria are small, likely episymbiotic organisms found across Earth’s ecosystems. Despite their prevalence, the distribution of CPR lineages across habitats and the genomic signatures of transitions among these habitats remain unclear. Here, we expand the genome inventory for Absconditabacteria (SR1), Gracilibacteria, and Saccharibacteria (TM7), CPR bacteria known to occur in both animal-associated and environmental microbiomes, and investigate variation in gene content with habitat of origin. By overlaying phylogeny with habitat information, we show that bacteria from these three lineages have undergone multiple transitions from environmental habitats into animal microbiomes. Based on co-occurrence analyses of hundreds of metagenomes, we extend the prior suggestion that certain Saccharibacteria have broad bacterial host ranges and constrain possible host relationships for Absconditabacteria and Gracilibacteria. Full-proteome analyses show that animal-associated Saccharibacteria have smaller gene repertoires than their environmental counterparts and are enriched in numerous protein families, including those likely functioning in amino acid metabolism, phage defense, and detoxification of peroxide. In contrast, some freshwater Saccharibacteria encode a putative rhodopsin. For protein families exhibiting the clearest patterns of differential habitat distribution, we compared protein and species phylogenies to estimate the incidence of lateral gene transfer and genomic loss occurring over the species tree. These analyses suggest that habitat transitions were likely not accompanied by large transfer or loss events but rather were associated with continuous proteome remodeling. Thus, we speculate that CPR habitat transitions were driven largely by availability of suitable host taxa and were reinforced by acquisition and loss of some capacities.

## INTRODUCTION

The Candidate Phyla Radiation (CPR) is a phylogenetically diverse clade of bacteria characterized by reduced metabolisms, potentially episymbiotic lifestyles, and ultrasmall cells (1–3). While the first high-quality CPR genomes were primarily from groundwater, sediment, and wastewater (4–6), subsequently genomes have been recovered from diverse environmental and animal-associated habitats, including humans. Intriguingly, from dozens of major CPR lineages, only three—Candidatus Absconditabacteria (formerly SR1), Gracilibacteria (formerly BD1-5 and GN02), and Saccharibacteria (formerly TM7)—are consistently associated with animal oral cavities and digestive tracts (7). The Saccharibacteria are perhaps the most deeply studied of all CPR lineages to date, likely due to their widespread presence in human oral microbiomes and association with disease states such as gingivitis and periodontitis (8, 9). On the other hand, Absconditabacteria and Gracilibacteria remain deeply undersampled, potentially due to their rarity in microbial communities or their use of an alternative genetic code that may confound some gene content analyses (4, 10, 11).

Absconditabacteria, Gracilibacteria, and Saccharibacteria are predicted to be obligate fermenters, dependent on other microorganisms (hosts) for components such as lipids, nucleic acids, and many amino acids (4, 6). Despite a generally reduced metabolic platform, CPR bacteria display substantial variation in their genetic capacities, even within lineages (12, 13). For example, some Gracilibacteria lack essentially all genes of the glycolysis and pentose-phosphate pathways and the tricarboxylic acid (TCA) cycle (14). In contrast to many CPR bacteria, soil-associated Saccharibacteria harbor numerous genes related to oxygen metabolism (15, 16). Pangenome analyses have shown genetic evidence for niche partitioning among Saccharibacteria from the same body site (17). However, the lack of comprehensive genomic sampling of these three CPR lineages across habitats, particularly from environmental biomes, has left unclear the full extent to which CPR gene inventories vary with habitat type, and, relatedly, the extent to which changes in metabolic capacities might have been reshaped during periods of environmental transition. Of particular interest is whether rapid gene acquisitions (e.g., via lateral gene transfer) or losses enabled habitat switches, or if these changes occurred gradually following habitat change.

The availability of suitable hosts may also drive the colonization of new environments by CPR bacteria (17). While there has been significant progress in characterizing the relationship between Saccharibacteria and Actinobacteria in the oral habitat (3, 18, 19), other CPR-host relationships remain unclear. Elucidation of environmental transitions among CPR lineages will require both thorough analysis of functional repertoires and a more comprehensive understanding of associations with other microorganisms. Here, we expand existing sampling of CPR genomes and their surrounding communities to examine patterns of distribution, abundance, and gene content in different microbiome types. We also make use of whole-community co-occurrence patterns to investigate the potential host range of CPR bacteria in their associated ecosystems. In combination, our analyses shed light on habitat shifts in three CPR lineages and the evolutionary processes likely underlying them.

## RESULTS

### Environmental diversity, phylogenetic relationships, and abundance patterns.

**FIG 1:**
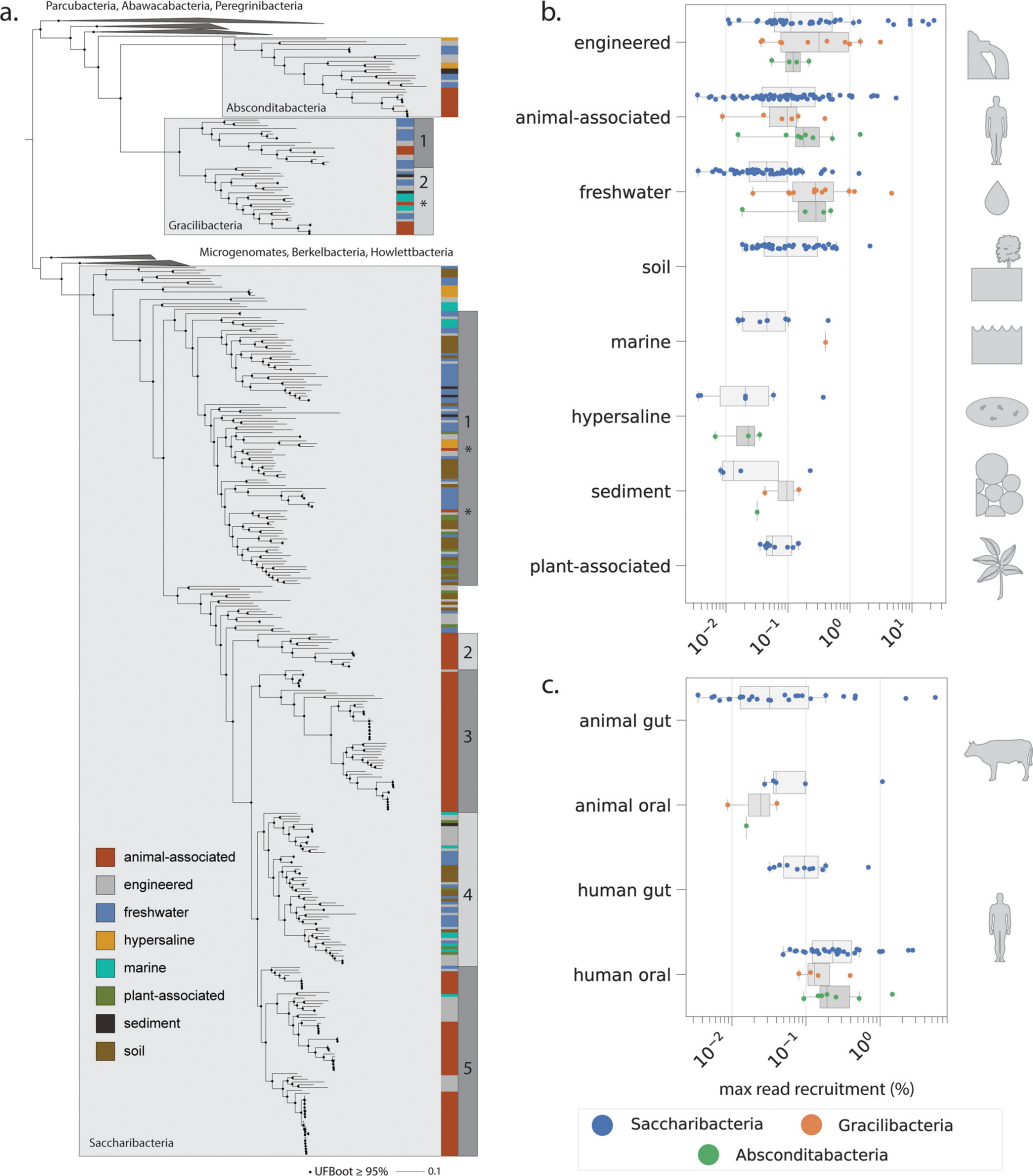
Phylogenetic and environmental patterns for the Absconditabacteria, Gracilibacteria, and Saccharibacteria. (a) Maximum-likelihood tree based on 16 concatenated ribosomal proteins (1,976 amino acids, LG+R10 model). Scale bar represents the average number of substitutions per site. Habitat of origin and phylogenetic subclade (where applicable) for each genome are indicated to the right of the tree. Asterisks indicate phylogenetic position of a subset of organisms derived from dolphin mouth metagenomes. (b and c) Percentage of reads per metagenomic sample mapping to individual genomes across environments (b) and body sites of humans and animals (c).

We gathered an environmentally comprehensive set of Absconditabacteria, Gracilibacteria, and Saccharibacteria by querying multiple databases for genomes assembled in previous studies and assembling new genomes from several additional metagenomic data sources (Materials and Methods and see Table S1 at https://doi.org/10.5281/zenodo.4560554) (2, 4–7, 10, 15–18, 20–83). Quality filtration of this curated genome set at ≥70% completeness and ≤10% contamination, plus subsequent dereplication at 99% average nucleotide identity (ANI), yielded a nonredundant set of 389 genomes for downstream analysis (see Table S1 at https://doi.org/10.5281/zenodo.4560554). Absconditabacteria and Gracilibacteria were less frequently sampled relative to Saccharibacteria, comprising only ∼7.5% and ∼10.8% of the total genome set, respectively. All three lineages were distributed across a broad range of microbiomes, encompassing various environmental habitats (freshwater, marine, soil, engineered, plant-associated, hypersaline) as well as multiple animal-associated microbiomes (oral and gut) (Fig. 1). Unlike animal-associated Gracilibacteria and Absconditabacteria genomes, which were recovered primarily from human and animal oral samples, animal-associated Saccharibacteria were found in both oral and gut samples.

We extracted 16 syntenic, phylogenetically informative ribosomal proteins from each genome to construct a CPR species tree and evaluate how habitat of origin maps onto phylogeny. Sequences from related CPR bacteria were used as outgroups for tree construction (Materials and Methods). The resolved topology supports monophyly of all three lineages and a sibling relationship between the two alternatively coded lineages, Absconditabacteria and Gracilibacteria (Fig. 1a; see also File S1 at https://doi.org/10.5281/zenodo.4560554), consistent with previous findings (13). For the Absconditabacteria, a single clade of organisms derived from animal-associated microbiomes was deeply nested within genomes from the environment. On the other hand, Gracilibacteria clearly formed two major lineages (GRA1 and -2), each with a small subclade comprised of animal-associated genomes (see Table S1 at https://doi.org/10.5281/zenodo.4560554). For Saccharibacteria, deeply rooting lineages were also almost exclusively of environmental origin (soil, water, sediment) and animal-associated genomes were strongly clustered into at least three independent subclades (Fig. 1a; see also Table S1 at https://doi.org/10.5281/zenodo.4560554). Two of these three subclades were exclusively composed of animal-associated sequences whereas one (SAC5) was a mixture of animal-associated, wastewater (potentially of human origin), and a few aquatic sequences. Intriguingly, for both Saccharibacteria and Gracilibacteria, a subset of organisms from the dolphin mouth (24) did not affiliate with those from terrestrial mammals/humans and instead fell within marine/environmental clades (indicated by asterisks in Fig. 1a). In primarily environmental clades (SAC1 and -4), genomes from soil, freshwater, engineered, and halophilic environments were phylogenetically interspersed, suggesting comparatively wide global distributions for these lineages. Exceptions to this pattern were two clades representing distinct hypersaline environments—a hypersaline lake and salt crust (66, 71).

We used read mapping to assess the abundance of Absconditabacteria, Gracilibacteria, and Saccharibacteria genomes in the samples from which they were originally reconstructed, focusing only on those organisms from short-read, whole-community sequencing experiments (Materials and Methods). In total, abundance calculation was possible for 297 of the 389 genomes (∼76%). Generally, these lineages of CPR bacteria are not dominant members of microbial communities (<1% of reads). However, they were relatively abundant in some engineered, animal-associated, and freshwater environments (Fig. 1b). In rare cases, CPR taxa comprised >10% of reads (Fig. 1b) and in a bioreactor (engineered) reached a maximum of ∼22% of reads. Gracilibacteria and Absconditabacteria attained read recruitment comparable to Saccharibacteria and were particularly abundant in some groundwater, engineered, and animal-associated habitats. In contrast to Saccharibacteria, Gracilibacteria and Absconditabacteria have so far been only minimally detected in soil and plant-associated microbiomes. We also compared abundance patterns across animal body sites. As expected based on extensive prior work (3, 8, 45), Saccharibacteria exhibited highest read recruitment in the human oral microbiome. However, these bacteria can also comprise a significant fraction of the sequenced DNA in exceptional gut/oral microbiomes from cows, pigs, and dolphins (Fig. 1c), in one case approaching 5% of reads (see Table S2 at https://doi.org/10.5281/zenodo.4560554). When detected, Saccharibacteria in the human gut were relatively rare, comprising a median of ∼0.1% of reads across samples.

### Patterns of co-occurrence constrain CPR host range across environments.

Despite recent progress made in experimentally identifying bacterial host ranges for oral Saccharibacteria, little is known about associations in other habitats. Abundance pattern correlations can be informative regarding associations involving obligate symbionts and their microbial hosts (39, 56); however, such analyses often rely on highly resolved time-series for statistical confidence. Here, we instead examine patterns of co-occurrence within samples to probe potential relationships between CPR bacteria and their microbial hosts. Given recent experimental evidence demonstrating the association of multiple Saccharibacteria strains with various Actinobacteria in the human oral microbiome (3, 18, 19, 45, 84), we predicted that Actinobacteria may be common hosts of Saccharibacteria in microbiomes other than the mouth and asked to what extent co-occurrence data supported this relationship.

**FIG 2:**
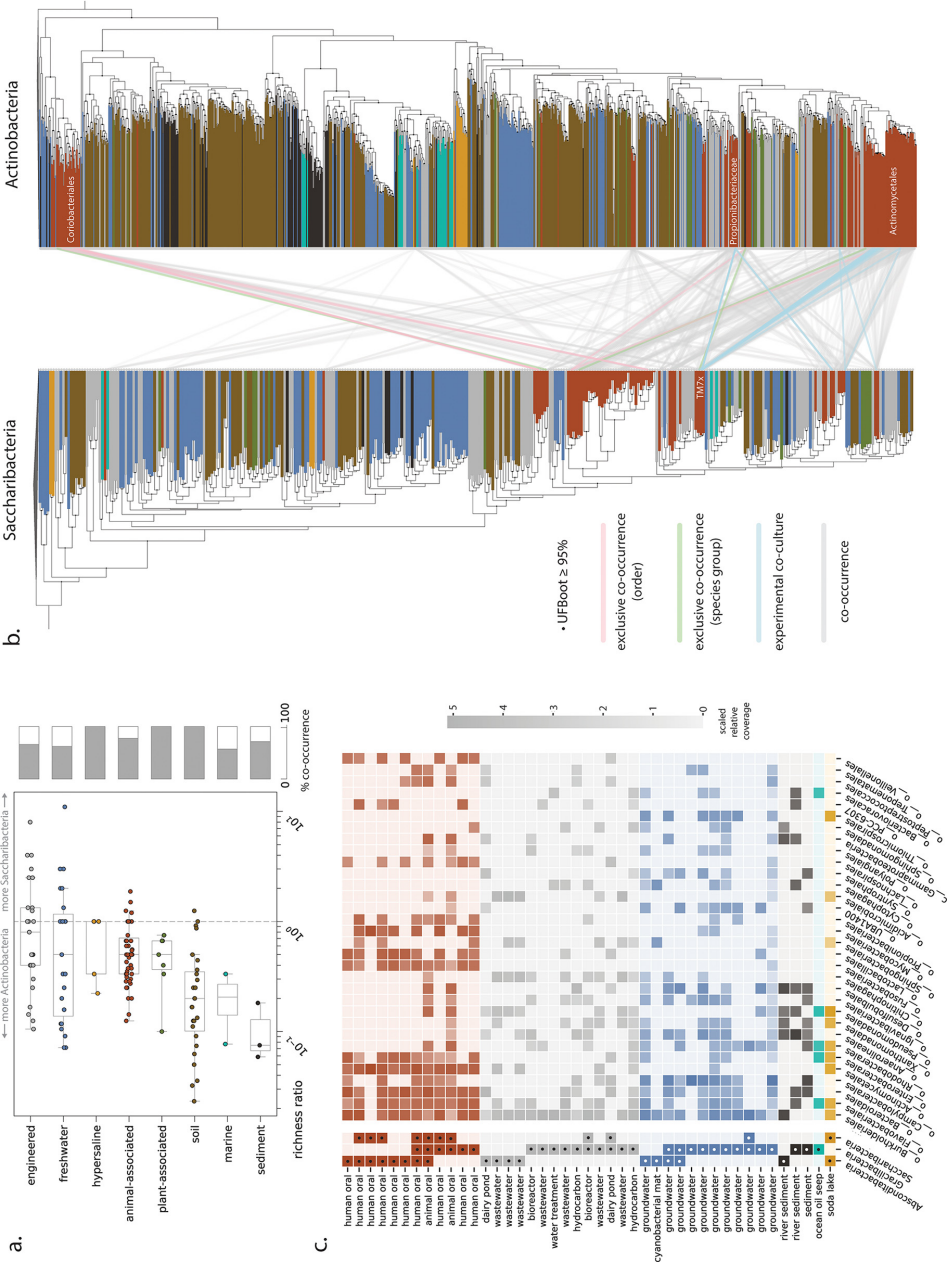
Patterns of co-occurrence between CPR and potential host lineages across environments. (a) Relative richness ratio, describing the ratio of distinct Saccharibacteria species groups to Actinobacteria species groups, for each sample and overall co-occurrence percentage across habitat categories. (b) Maximum-likelihood trees for Saccharibacteria and Actinobacteria based on ribosomal protein S3 sequences extracted from all source metagenomes. Co-occurrence patterns are shown only for species groups derived from animal-associated metagenomes. (c) Community composition (using GTDB taxonomy for non-CPR bacteria) for metagenomic samples containing Absconditabacteria and Gracilibacteria. Cells with dots indicate only presence, whereas those without dots convey log-scaled, normalized relative coverage information. Only potential host lineages present in 8 or more samples are shown.

We first identified all ribosomal protein S3 (rpS3) sequences from Actinobacteria and Saccharibacteria in the source metagenomes probed in this study for abundance patterns (Fig. 1b and c). rpS3 sequences from all samples were clustered into “species groups” (Materials and Methods). We observed that species groups from Actinobacteria and Saccharibacteria frequently cooccurred in the soil and plant-associated microbiomes as well as several hypersaline microbiomes (Fig. 2a). On the other hand, co-occurrence of the two lineages was less frequent in engineered and freshwater environments relative to other environments. Surprisingly, only ∼78% of animal-associated samples containing Saccharibacteria also contained Actinobacteria at abundances high enough to be detected (Fig. 2a). The absence of Actinobacteria in the remaining animal-associated samples was confirmed with an additional marker gene, ribosomal protein L6 (rpL6) (Materials and Methods). Assemblies with well-sampled Saccharibacteria yet no detectable Actinobacteria could suggest that Saccharibacteria have alternative hosts in these samples or are able to (at least periodically) live independently. Alternatively, the lack of Actinobacteria rpS3/rpL6 in these samples could be the result of poor sequence assembly, e.g., due to population heterogeneity or low coverage.

For samples where both Saccharibacteria and Actinobacteria marker genes were detectable, we computed a “relative richness” metric describing the ratio of distinct Saccharibacteria species groups to Actinobacteria species groups. In most animal-associated microbiomes, Actinobacteria were more species rich (lower richness ratios), as expected if individual Saccharibacteria can associate with multiple hosts (Fig. 2a). Greater species richness of Actinobacteria than of Saccharibacteria was also observed for many plant-associated, soil, engineered, and freshwater microbiomes. However, some engineered and freshwater samples had richness ratios equal to (equal richness) or greater than 1 (i.e., Saccharibacteria more species rich) (Fig. 2a). Specifically, we observed that several metagenomes from engineered and freshwater environments contained anywhere from 1 to 11 Saccharibacteria species but only one detectable Actinobacteria species (see Table S3 at https://doi.org/10.5281/zenodo.4560554). Thus, if Actinobacteria serve as hosts for Saccharibacteria in these habitats, there may be both exclusive associations and associations linking multiple Saccharibacteria species with a single Actinobacteria host species.

We next tested for more specific possible associations in the animal microbiome, reasoning that if Actinobacteria are common hosts for Saccharibacteria, then exclusive co-occurrence of a particular Saccharibacteria species with a singular Actinobacteria species within a sample might suggest an interaction *in vivo*. We mapped all pairs of Saccharibacteria and Actinobacteria species that cooccurred within a single sample onto species trees constructed from recovered rpS3 sequences (Fig. 2b), including 22 Saccharibacteria-Actinobacteria pairs reported in previous experimental studies (see Table S4 at https://doi.org/10.5281/zenodo.4560554). In three cases, we found that individual metagenomic samples contained only one assembled Saccharibacteria species group and one Actinobacteria species group (“exclusive co-occurrence - species group,” Fig. 2b; see also Table S3 at https://doi.org/10.5281/zenodo.4560554). Two of these cases involved Actinobacteria from the order Actinomycetales, from which multiple Saccharibacteria hosts have already been identified (85). We also noted exclusive species-level co-occurrence of a Saccharibacteria species group from the human gut and an Actinobacteria species group from the order Coriobacteriales (see Table S3 at https://doi.org/10.5281/zenodo.4560554). In an additional seven cases, one Saccharibacteria species group occurred with multiple Actinobacteria species groups of the same order-level classification based on rpS3 gene profiling (“exclusive co-occurrence - order,” Fig. 2b; see also Table S3 at https://doi.org/10.5281/zenodo.4560554). Five of the seven instances involved pairs of Saccharibacteria and Coriobacteriales from termite and swine gut metagenomes. Thus, unlike in human oral environments, Coriobacteriales may serve as hosts for Saccharibacteria in gut environments of multiple animal species. More generally, we also observed that Saccharibacteria from the same phylogenetic clade had predicted relationships to phylogenetically unrelated Actinobacteria (Fig. 2b), consistent with previous experimental observations for individual species (45).

Compared to Saccharibacteria, host relationships for Gracilibacteria and Absconditabacteria have received little attention. There are preliminary indications that Absconditabacteria may associate with members of the Fusobacteria or Firmicutes in the oral microbiome (45) or the gammaproteobacterium *Halochromatium* in certain salt lakes (86). We thus explored co-occurrence patterns in microbial communities containing Absconditabacteria and Gracilibacteria, attempting to further constrain possible host taxa. In animal- and human-associated microbiomes, bacteria from several lineages, including Fusobacteria (Fig. 2c), were relatively abundant in nearly all samples that contained Absconditabacteria. Members of the Chitinophagales, Pseudomonadales, and Acidimicrobiales were detected in high abundance in three wastewater samples from similar treatment plants (42) and one dairy pond sample containing Absconditabacteria (Fig. 2c; see also Table S5 at https://doi.org/10.5281/zenodo.4560554). No clear patterns of potential host co-occurrence were observed for Gracilibacteria, with the exception of the proteobacterial order Campylobacterales, which cooccurred in 8 of 10 groundwater samples where Gracilibacteria were found (Fig. 2c). Across all habitat types, only members of the order Burkholderiales (a large order of Gammaproteobacteria) consistently cooccurred with Gracilibacteria; however, these organisms were also abundant in a number of samples without detectable Gracilibacteria, weakening the potential association.

Among the least complex communities that contained Absconditabacteria were cyanobacterial mats from a California river network, where dominant cyanobacterial taxa accounted for ∼60 to 98% of relative abundance (34). To complement the above co-occurrence analyses, we reanalyzed 22 published metagenomes representing spatially separated mats and discovered that Absconditabacteria were detectable in 12 of them at various degrees of coverage (0.12× to 37×). As noted previously, also present in the mats were members of the phyla Bacteroidetes, Betaproteobacteria, and Verrucomicrobia (34). Correlation of read coverage profiles across mats provided moderate support for the association of Absconditabacteria and Bacteroidetes. Specifically, many of the strongest species-level correlations, including five of the top 10, involved Bacteroidetes (see Table S6 at https://doi.org/10.5281/zenodo.4560554).

### Gene content of Absconditabacteria, Gracilibacteria, and Saccharibacteria.

**FIG 3:**
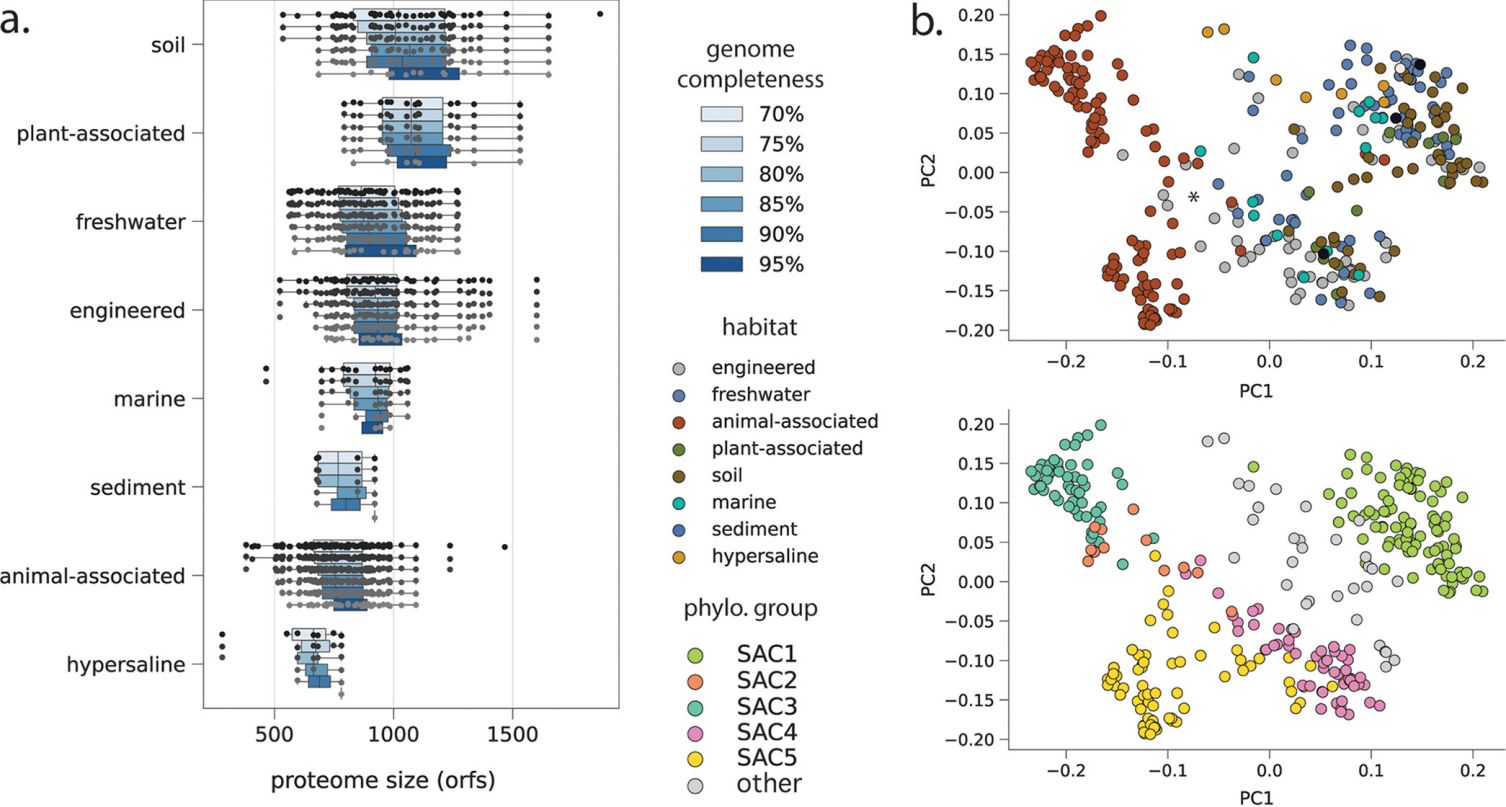
Proteome characteristics for Saccharibacteria. (a) Predicted proteome size (open reading frame count) at increasing genome completeness thresholds. (b) Overall proteome similarity among Saccharibacteria from different habitat categories (top panel) and phylogenetic clades (bottom panel). PCoAs were computed from presence/absence profiles of all protein clusters with 5 or more member sequences. The primary (PC1) and secondary (PC2) principal coordinates explained 12% and 8% of variance, respectively.

We next examined how gene content of these CPR lineages varied across environments. We first compared the predicted proteome sizes of these bacteria across habitats, taking into consideration differing degrees of genome completeness. This analysis revealed that genomes from soil and the rhizosphere (plant associated) have on average larger predicted proteomes than those from animal-associated environments (Fig. 3a). Saccharibacteria from hypersaline environments appear to have the smallest predicted proteomes, although the limited number of high-quality genomes in this category currently limits a firm conclusion. We observed some evidence for variance in predicted proteome sizes among Absconditabacteria and Gracilibacteria, including potentially smaller predicted proteomes among animal-associated Gracilibacteria (see Fig. S1 at https://doi.org/10.5281/zenodo.4560554). Additional high-quality genomes will be required to confirm this trend.

To examine overall proteome similarity as a function of habitat type, we employed a recently developed protein-clustering approach that is agnostic to functional annotation (12) (Materials and Methods; see also Table S7 at https://doi.org/10.5281/zenodo.4560554). Among Saccharibacteria, principal-coordinate analysis (PCoA) of presence/absence profiles for all protein families with 5 or more members yielded a primar*y* axis of variation (∼12% variance explained) that distinguished animal-associated Saccharibacteria from environmental or plant-associated ones and a secondar*y* axis (∼8% variance explained) that distinguished between phylogenetic clades (SAC1 to -3 versus SAC4 and SAC5). We did not observe strong clustering of Saccharibacteria by specific environmental biome, consistent with the interspersed nature of their phylogenetic relationships (Fig. 1a and Fig. 3b). Notably, several SAC5 genomes from wastewater have protein family contents that are intermediate between those of animal-associated Saccharibacteria and Saccharibacteria from the large environmental clade (indicated by an asterisk in Fig. 3b). This finding may indicate selection within the engineered environments for variants introduced from human waste. PCoAs of predicted proteome content among Absconditabacteria and Gracilibacteria generally showed that, with the exception of dolphin-derived genomes, animal-associated lineages are also distinct from their relatives from environmental biomes (see Fig. S2 at https://doi.org/10.5281/zenodo.4560554). Overall, our results indicate that the CPR lineages examined here have predicted proteomes whose content and size vary substantially with their environment. This is particularly evident for animal-associated Saccharibacteria, which are notably dissimilar in their protein family content from environmental counterparts.

**FIG 4:**
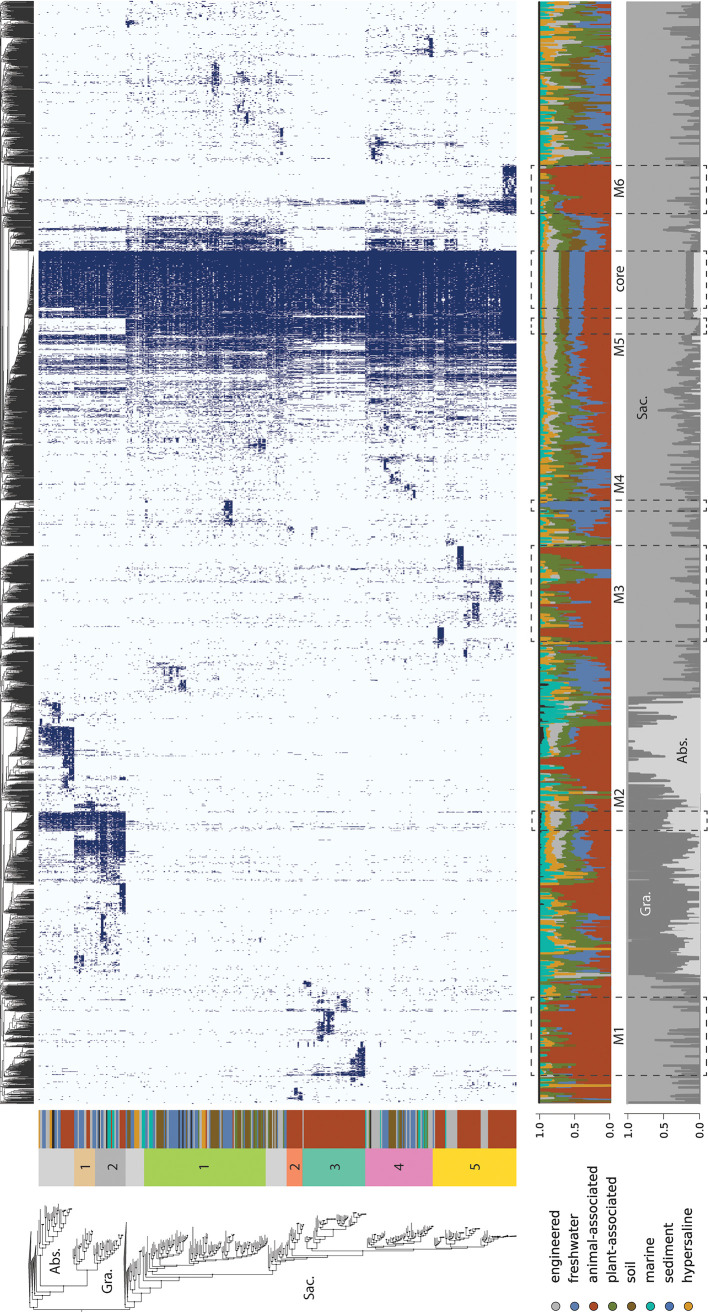
Phylogenetic and environmental distribution of protein families recovered among CPR bacteria. (Upper panel) Presence/absence profiles for protein families with 5 or more members, with shaded cells indicating presence and light cells indicating absence. Columns represent protein families, hierarchically clustered by similarity in distribution across the genome set. Rows correspond to genomes, ordered by their phylogenetic position in the species tree (left). Abbreviations: Abs., Absconditabacteria; Gra., Gracilibacteria; Sac., Saccharibacteria. (Lower panels) Percentage of genomes encoding individual protein families that belonged to broad habitat groups (top) or taxonomic groups (bottom). Modules of protein families indicated in the text are represented by dashed lines (M1 to -6 and “core”).

To further examine the distinctions evident in the PCoA, we arrayed presence/absence information for each protein family and hierarchically clustered them based on their distribution patterns across all three CPR phyla. This strategy allowed us to explore specific protein family distributions and to test for groups of cooccurring protein families (modules) that are common to bacteria from a single lineage or are shared by most bacteria from one or more CPR lineages. We first observed one large module that is generally conserved across all genomes. This module is comprised of families for essential cellular functions such as transcription, translation, cell division, and basic energy generating mechanisms (Fig. 4, “core”).

The protein family analysis also revealed multiple modules specific to Gracilibacteria and Absconditabacteria and modules shared by both lineages but not present in Saccharibacteria, paralleling their phylogenetic relationships (Fig. 1a and Fig. 4). Of the ∼70 families shared only by Gracilibacteria and Absconditabacteria (M2, Fig. 4), nearly half had no KEGG annotation at the thresholds employed. One family shared by these phyla but not in Saccharibacteria is the ribosomal protein L9, which supports prior findings on the composition of Saccharibacteria ribosomes (31). The remaining families also include two that were fairly confidently annotated as the DNA mismatch repair proteins, MutS and MutL (fam01378 and fam00753), nicking endonucleases involved in correction of errors made during replication (87) (see Table S7 at https://doi.org/10.5281/zenodo.4560554). Despite the generally wide conservation of these proteins among Bacteria, we saw no evidence for the presence of either enzyme in Saccharibacteria, suggesting that aspects of DNA repair may vary in this group relative to other CPR bacteria. We recovered a module of approximately 60 proteins highly conserved among the Saccharibacteria and only rarely encoded in the other lineages (M5, Fig. 4). This module contained several protein families confidently annotated as core components of glycolysis and the pentose phosphate pathway, including three enzymes present in almost all CPR bacteria (13): glyceraldehyde 3-phosphate dehydrogenase, (GAPDH), triosephosphate isomerase (TIM), and phosphoglycerate kinase (PGK). These results indicate that Gracilibacteria and Absconditabacteria may have extremely patchy, if not entirely lacking, components of core carbon metabolism, even when a high-quality genome set is considered.

For all three lineages of CPR, we also observed numerous small modules with narrow distributions. To test whether these modules represent functions differentially distributed among organisms from different habitats, we computed ratios describing the incidence of each protein family in genomes from one habitat compared to those from all other habitats (Materials and Methods). Enriched families were defined as those with ratios of ≥5, whereas depleted families were defined as those that were encoded by <10% of genomes in a given habitat but ≥50% of genomes from other habitats. To account for the fact that small families might appear to be differentially distributed due to chance alone, we also stipulated that comparisons be statistically significant (*P* ≤ 0.05, two-sided Fisher’s exact test corrected for multiple comparisons).

Using this approach, we identified 926 families that were either enriched (*n* = 872) or depleted (*n* = 54) in genomes from one or more broad habitat groups. We identified 45 families enriched in Absconditabacteria from animal-associated environments relative to those from environmental biomes. The majority of these families were either poorly functionally characterized or entirely without a functional annotation at the thresholds employed. Similarly, families enriched in animal-associated Gracilibacteria relative to environmental counterparts were primarily unannotated; among those families with confident annotations was a family likely encoding a phosphate:Na^+^ symporter (fam04488) and a putative membrane protein (fam06579). Intriguingly, 6 families were coenriched in both animal-associated Gracilibacteria and Absconditabacteria, suggesting that these sibling lineages might have acquired or retained a small complement of genes that are important in adaptation to animal habitats or their associated bacteria.

Animal-associated Saccharibacteria, on the other hand, encoded 417 unique families that were exclusive or highly enriched relative to those from other habitats. Enriched families largely fell into three major groups (M1, M3, M6 [Fig. 4]), and the large majority of them, particularly among modules with narrow, lineage-specific distributions, were without functional annotations. However, our analysis also revealed some protein families with broader distributions across multiple clades of animal-associated Saccharibacteria (Fig. 4). Here, among families with functional annotations, we found several apparently involved in the transport of amino acids and dicarboxylates that were highly enriched (ratios ranging from 10.7 to 112.9) in the majority of animal-associated Saccharibacteria (52 to 58% of genomes across clades) (see Table S8 at https://doi.org/10.5281/zenodo.4560554). Two of these families, corresponding to a putative amino acid transport permease and substrate-binding protein (fam00393 and fam11477, respectively), were colocated in some genomes along with an ATP-binding protein (subset of fam00001), suggesting that they may function together to take up amino acids. We also recovered several other functions that were previously predicted to be enriched based on analysis of a smaller set of animal-associated Saccharibacteria (7), including phosphoglycerate mutase, glycogen phosphorylase, and a uracil-DNA glycosylase (ratio 8.3 to 33.5). Lastly, we found that one family encoding the CRISPR-associated protein csn1/cas9 (fam00646) was also enriched among animal-associated genomes (ratio ∼12.4 among 28 genomes), consistent with the suggestion that some Saccharibacteria likely acquired their viral defense systems after colonizing animals (see Table S8 at https://doi.org/10.5281/zenodo.4560554) (7).

We identified multiple families that are either enriched or depleted in animal-associated Saccharibacteria that were functionally related to oxidative stress (see Table S8 at https://doi.org/10.5281/zenodo.4560554). Among enriched families, one (fam00662) set was mostly annotated with low confidence as rubrerythrin, a family of iron-containing proteins generally involved in detoxification of peroxide (88). Member sequences of this family were present in over a third of animal-associated Saccharibacteria and were highly enriched relative to environmental genomes (fold-enrichment ratio of 36.2), suggesting that acquisition may have conferred an adaptive benefit in the gut and/or oral cavity. In contrast, we also observed that animal-associated Saccharibacteria were significantly depleted in another family confidently annotated as a Fe-Mn family superoxide dismutase (fam01569) and likely involved in radical detoxification. Animal-associated lineages were also strongly depleted for the genes comprising the cytochrome *o* ubiquinol oxidase operon (fam00281, fam00112, fam01347, fam00624, and fam10494), with very few, if any, animal-associated genomes and more than 50% of environmental genomes harboring each of the five genes. This operon has been previously suggested to confer an advantage in aerophilic environments like soil through detoxification (6) or use of oxygen (15, 16).

Among genomes from environmental biomes, we identified a module of approximately 100 protein families, also primarily without functional annotation, that were associated with a subclade of Saccharibacteria recently reconstructed from metagenomes of freshwater lakes and glacier ice (M4, Fig. 4) (62, 89). Notably, among the most widespread families in this module was one in which sequences were annotated as bacteriorhodopsin with low confidence (fam11249). Further analysis indicated that these sequences fall within the bacterial/archaeal type 1 rhodopsin clade and contain both the retinal-binding lysine associated with light sensitivity and a DTS motif (see Fig. S3 at https://doi.org/10.5281/zenodo.4560554), suggesting that they may function as proton pumps (90, 91). Distinct rhodopsin sequences were also recovered in the genomes of environmental Absconditabacteria (NDQ motif) and Gracilibacteria (DTE motif), although they were not statistically enriched (see Fig. S3 at https://doi.org/10.5281/zenodo.4560554). Genomes of soil-associated Saccharibacteria were enriched for nearly 130 protein families largely without strong functional annotations (Fig. 4; see also Table S8 at https://doi.org/10.5281/zenodo.4560554). Despite their small proteome sizes, Saccharibacteria from hypersaline environments were only statistically depleted in about 15 families at the thresholds employed here. Sequence files for all protein families are provided in File S2 in the supplemental material at https://doi.org/10.5281/zenodo.4560554.

### Evolutionary processes shaping proteome evolution.

**FIG 5:**
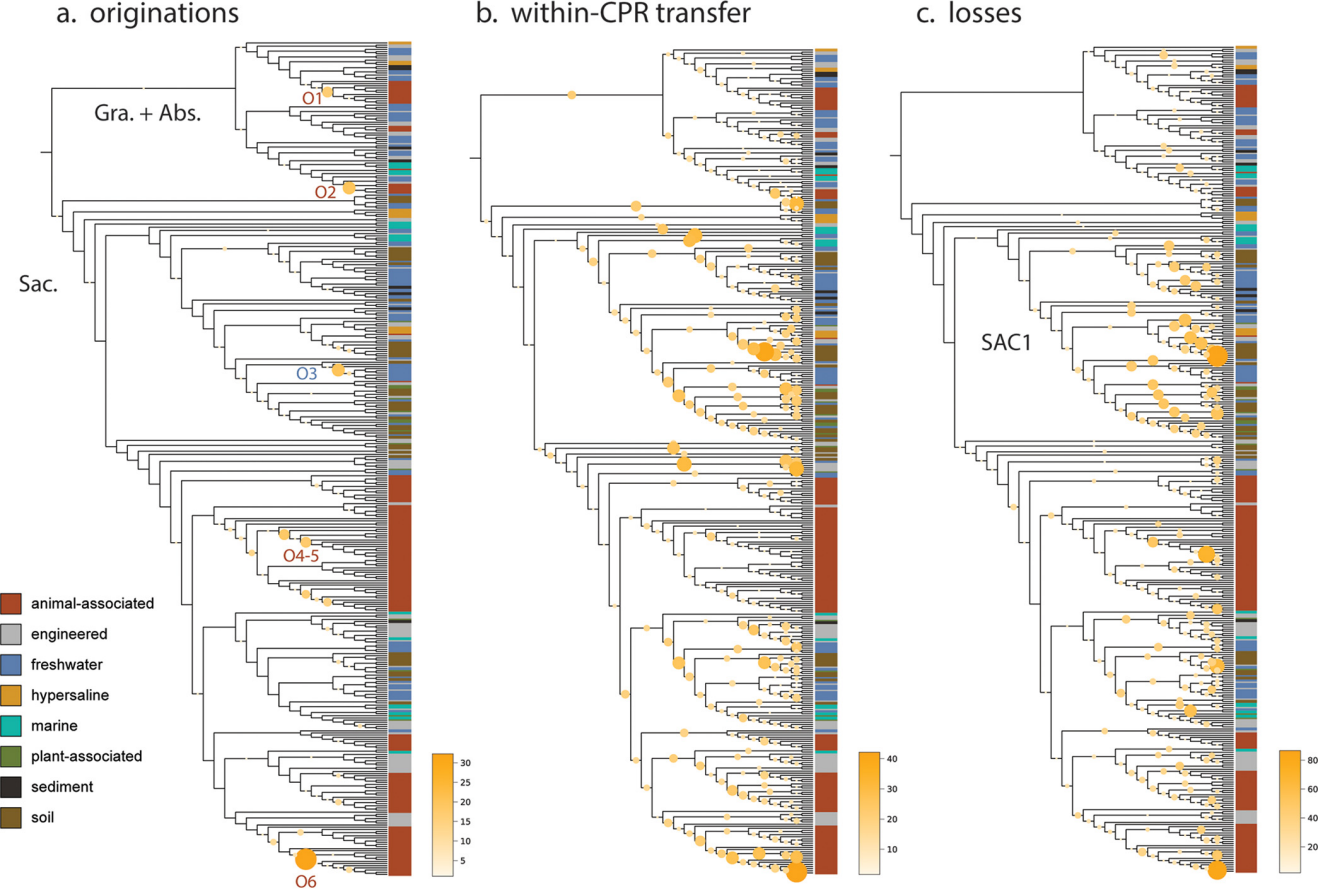
Evolutionary processes shaping proteome evolution in three lineages of CPR bacteria. Each panel displays the species tree from Fig. 1a in cladogram format. The size and color of circles mapped onto interior branches represent the cumulative number of originations (defined as either lateral transfer from outside the lineages examined here or *de novo* evolution) (a), transfer among the three CPR lineages included here (b), and genomic losses predicted to occur on that branch for all 902 differentially distributed families where gene-species tree reconciliation was possible (c). Abbreviations: Abs., Absconditabacteria; Gra., Gracilibacteria; Sac., Saccharibacteria. SAC1 indicates a monophyletic clade of Saccharibacteria referenced in the text.

The observation that some differentially distributed traits among CPR bacteria were apparently lineage specific, whereas others were more widespread, motivated us to examine the relative contributions of gene transfer and loss to proteome evolution. To do so, we first inferred unrooted, maximum-likelihood phylogenies for the sequences in each protein family that was differentially distributed and then compared these phylogenies to the previously reconstructed species tree (Materials and Methods). For each family, the likelihood of transfer and loss events on each branch of the species tree was then estimated using a probabilistic framework that takes into consideration genome incompleteness, variable rates of transfer and loss, and uncertainty in gene tree reconstruction (92, 93). The results of this analysis reveal relatively few instances of originations, defined as lateral transfer from outside the three lineages of CPR bacteria or *de novo* evolution (“originations,” Fig. 5). In the Absconditabacteria and Gracilibacteria, gene-species tree reconciliation revealed that small modules of families of mostly hypothetical proteins were acquired near the base of animal-associated clades (O1 and O2, Fig. 5; see also Table S1 at https://doi.org/10.5281/zenodo.4560554). On the other hand, in Saccharibacteria, originations were primarily associated with shallower subclades of animal-associated (and, in one case, freshwater) genomes (O3 to O6, Fig. 5; see also Table S1 at https://doi.org/10.5281/zenodo.4560554). These findings generally corresponded with the distribution of small, highly enriched modules of largely hypothetical proteins (Fig. 4) and suggest that the distribution of these modules is best explained by lineage-specific acquisition events of relatively few genes at one time, rather than large acquisition events at deeper nodes. Intriguingly, one subclade of animal-associated Saccharibacteria had the highest incidence of originations of all groups in our analysis (O6, Fig. 5; see also Table S1 at https://doi.org/10.5281/zenodo.4560554), suggesting that these genomes may be phylogenetic “hot spots” for transfer.

While origination events were relatively infrequent in all three CPR lineages, instances of within-CPR transfer and loss were very frequent and dispersed across most interior branches of the tree (Fig. 5). Notably, we detected sporadic losses across internal branches, which is inconsistent with a major gene loss event at the time of adaptation to animal-associated habitats. Surprisingly, we noticed that genomes of non-animal-associated Saccharibacteria, particularly those from the SAC1 clade, displayed substantial patterns of loss despite their relatively large proteome sizes. Thus, losses in these environmental lineages were possibly balanced by lateral transfer events over the course of evolution.

## DISCUSSION

Here, we expand sampling of genomes from the Absconditabacteria, Gracilibacteria, and Saccharibacteria, particularly from environmental biomes. The basal positioning of environmental clades in phylogenetic reconstructions provides strong support for the hypothesis that these lineages originated in the environment (Fig. 1a) and potentially migrated into humans and terrestrial animals via consumption of groundwater (2, 7). Unlike the Absconditabacteria, which appear to have transitioned only once into animal oral cavities and guts, our phylogenetic evidence suggests that Gracilibacteria may have undergone multiple transitions into the animal microbiome in unique phylogenetic clades. In the Saccharibacteria, phylogenetically interspersed environmental and oral/gut Saccharibacteria could reflect independent migrations into the animal environment, consistent with previous work on smaller genome sets (7). Alternatively, this pattern may reflect lineage-specific reversion to environmental niches in some clades (Fig. 1a). We also show preliminary evidence for small lineages of Gracilibacteria and Saccharibacteria that appear to have colonized the dolphin mouth separately from those that colonized the oral environments of terrestrial animals (Fig. 1a). Previous work showing the clear distinction between marine mammal microbiomes and their surrounding seawater/prey supports the idea that these CPR bacteria are likely legitimate members of the dolphin oral microbiome, rather than contamination (94).

Currently, the mechanisms that enable environmental transition among CPR bacteria are unknown. Several observations, including that CPR host-pairs may be taxonomically distinct between oral and gut habitats, raise the question of whether habitat transitions among CPR bacteria involve comigration with their hosts or the acquisition of new hosts. The finding that single CPR species cooccur with a single Actinobacteria species, or several closely related ones, in multiple animal-associated metagenomes contributes further evidence that these associations may be flexible and phylogenetically diverse rather than highly evolutionarily conserved (45). Supporting this, some laboratory strains of oral Saccharibacteria can adapt to new hosts after periods of living independently (95). The lack of evidence for lateral gene transfer between experimentally profiled pairs (7) also suggests that some CPR-host pairs may have established fairly recently.

Host associations for Absconditabacteria, Gracilibacteria, and environmental Saccharibacteria are largely unknown. However, this is changing quickly—for example, a very recent paper demonstrated the coculture of Saccharibacteria and two species of Gordonia (Actinobacteria) from wastewater foam (96). Similarly, changes in abundance over a sample series from a bioreactor system treating thiocyanate were recently used to suggest that Microbacterium ginsengisoli may serve as a host for a cooccurring Saccharibacteria bacterium (56). One Absconditabacteria lineage (*Vampirococcus*) has been predicted to have a host from the Gammaproteobacteria (86), and one Gracilibacteria was suggested to have a *Colwellia* host based on a shared repeat protein motif (14). Given the scant information about possible hosts for these CPR bacteria, especially for Absconditabacteria and Gracilibacteria, the patterns of co-occurrence we report for specific organisms provide starting points for host identification via targeted coisolation.

To evaluate to what extent changes in gene content are associated with habitat transition, we first established core gene sets. These indicated that overall proteome size and content differed between environmental and animal-associated Saccharibacteria, and to some extent Gracilibacteria. Despite overall smaller proteome size, we identified a large number of protein families that were highly enriched among animal-associated CPR bacteria from all three lineages. The most striking capacities involve amino acid transport, oxidative stress tolerance, and viral defense, which may enable use of habitat-specific resources or tolerance of habitat-specific stressors. These findings complement previous evidence that prophages are enriched in animal-associated Saccharibacteria relative to environmental counterparts (17).

Only three lineages of CPR bacteria (of potentially dozens) have been consistently recovered in the animal-associated microbiome. Given the enormous diversity of CPR bacteria in drinking water (2), there has likely been ample opportunity for various taxa to disperse into the mouths of terrestrial animals; however, establishment and persistence of these bacteria may have been limited by the absence of a suitable host in oral and gut environments. Thus, we predict that other CPR bacteria—including those from the large Microgenomates and Parcubacteria lineages—have hosts that are infrequent or transient members of the animal microbiome or have insufficient ability to “adapt” to new hosts upon contact. For example, formation of new associations may be limited by the specificity of pili involved in host interaction or proteins involved in attachment (1, 2, 17).

It is also interesting to compare processes of habitat transition in CPR bacteria with those proposed for other bacteria and for archaea. Our results suggest that Saccharibacteria (and potentially Gracilibacteria) from the human/animal microbiome have smaller genome sizes than related, deeper-branching lineages of environmental origin. This pattern is also apparent for other, free-living groups adapted to the animal microbiome from the environment, like the Elusimicrobia (97) and intracellular symbionts of insects (98). However, in contrast to findings for Elusimicrobia, where host-associated lineages have common patterns of loss of metabolic capacities compared to relatives from nonhost environments (97), patterns of gene loss in animal-associated CPR bacteria appear to be heterogeneous and lineage specific. One possibility is that gene loss in CPR bacteria is primarily modulated by strong dependence on host bacteria, whose capacities may vary substantially, rather than by adaptation to the relatively stable, nutrient-rich animal habitat that likely shaped evolution of some non-CPR bacteria.

Changes in gene content could enable, facilitate, or follow habitat transitions. Our evolutionary reconstructions revealed that habitat-specific differences in gene content are more likely the product of combinations of intra-CPR transfer and loss rather than major acquisition events at time of lineage divergence. Thus, modules enriched in specific lineages were probably acquired via lateral transfer after habitat transition, suggesting that proteome remodeling has been continuous in CPR bacteria over evolutionary time. As such, the processes shaping CPR lineage evolution share both similarities with and differences from those predicted for other microbes, including Haloarchaeota (99) and ammonia-oxidizing lineages of Thaumarchaeota (92, 100), where both large lateral transfer events and gradual patterns of gene loss, gain, and duplication worked together to shape major habitat transitions.

### Conclusion.

Overall, our findings highlight factors associated with habitat transitions in three CPR lineages that occur in both human/animal and environmental microbiomes. We expand the evidence for niche-based differences in protein content (7, 17) and identify a large set of protein families that could guide future studies of CPR symbiosis. Furthermore, patterns of co-occurrence may inform experiments aiming to cocultivate CPR bacteria and their hosts. Our analyses point to a history of continuous genome remodeling accompanying transition into human/animal habitats, rather than rapid gene gain/loss around the time of habitat switches. Thus, habitat transitions in CPR may have been primarily driven by the availability of suitable hosts and reinforced by acquisition and/or loss of genetic capacities. These processes may be distinct from those shaping transitions in other bacteria and archaea that are not obligate symbionts of other microorganisms.

## MATERIALS AND METHODS

### Genome database preparation and curation.

To compile an environmentally comprehensive set of genomes from the selected CPR lineages, we first queried four genomic information databases—GTDB (https://gtdb.ecogenomic.org/), NCBI assembly (https://www.ncbi.nlm.nih.gov/assembly), PATRIC (https://www.patricbrc.org/), and IMG (https://img.jgi.doe.gov/)—for records corresponding to the Absconditabacteria, Gracilibacteria, and Saccharibacteria genomes. Genomes gathered from these databases were combined with those drawn from several recent publications as well as genomes newly binned from metagenomic samples of sulfidic springs, an advanced treatment system for potable reuse of wastewater, human saliva, cyanobacterial mats, fecal material from primates, baboons, pigs, goats, cattle, and rhinoceros, several deep subsurface aquifers, dairy-impacted groundwater and associated enrichments, multiple bioreactors, soil, and sediment (see Table S1 at https://doi.org/10.5281/zenodo.4560554). Assembly, annotation, and binning procedures followed those from Anantharaman et al. (33). In some cases, manual binning of the alternatively coded Absconditabacteria was aided by a strategy in which a known Absconditabacteria gene was blasted against predicted metagenome scaffolds to find “seed” scaffolds, whose coverage and GC profile were used to probe remaining scaffolds for those with similar characteristics. For newly binned genomes, genes were predicted for scaffolds >1 kb using prodigal (“meta” mode) and annotated using USEARCH against the KEGG, UniProt, and UniRef100 databases. Bins were “polished” by removing potentially contaminating scaffolds with phylogenetic profiles that deviated from consensus taxonomy at the phylum level. One genome was further manually curated to remove scaffolding errors identified by read mapping, following the procedures outlined in reference 101.

We removed exact redundancy from the combined genome set by identifying identical genome records and selecting one representative for downstream analyses. We then computed contamination and completeness for the genome set using a set of 43 marker genes sensitive to described lineage-specific losses in the CPR bacteria (31, 33) using the custom workflow in CheckM (102). Results were used to secondarily filter the genome set to those with ≥70% of the 43 marker genes present and ≤10% of marker genes duplicated. The resulting genomes were then dereplicated at 99% ANI using dRep (-sa 0.99 -comp 70 -con 10) (103), yielding a set of 389 nonredundant genomes from a starting set of 868. Existing metadata were used to assign both “broad” and “narrow” habitat of origin for each nonredundant genome. The “engineered” habitat category was defined to include human-made or industrial systems like wastewater treatment, bioreactors, and water impacted by farming/mining. Curated metadata, along with accession/source information for each genome in the final set, are available in Table S1 at https://doi.org/10.5281/zenodo.4560554. All newly binned genomes are available through Zenodo (see “Data and software availability” below).

### Functional annotation and phylogenomics.

We predicted genes for each genome using prodigal (“single” mode), adjusting the translation table (*-g 25*) for Gracilibacteria and Absconditabacteria, which are known to utilize an alternative genetic code (10, 11). Predicted proteins were concatenated and functionally annotated using kofamscan (104). Results with an E value of ≤1e−6 were retained and subsequently filtered to yield the highest-scoring hit for each individual protein.

To create a species tree for the CPR groups of interest, functional annotations from kofamscan were queried for 16 syntenic ribosomal proteins (rp16). Marker genes were combined with those from a set of representative sequences of major, phylogenetically proximal CPR lineages (13). Sequences corresponding to each ribosomal protein were separately aligned with MAFFT and subsequently trimmed for phylogenetically informative regions using BMGE (*-m BLOSUM30*) (105). We then concatenated individual protein alignments, retaining only genomes for which at least 8 of 16 syntenic ribosomal proteins were present. A maximum-likelihood tree was then inferred for the concatenated rp16 (1,976 amino acids) set using ultrafast bootstrap and IQ-TREE’s extended Free-Rate model selection (*-m MFP -st AA -bb 1000*) (106). The maximum-likelihood tree is available as File S1 at https://doi.org/10.5281/zenodo.4560554. The tree and associated metadata were visualized in iTOL (107) where well-supported, monophyletic subclades were manually identified within Gracilibacteria and Saccharibacteria for use in downstream analysis.

### Abundance analysis.

To assess the global abundance of Absconditabacteria, Gracilibacteria, and Saccharibacteria, we manually compiled the original read data associated with each genome in the analysis set, where available. We included only those genomes from short-read, shotgun metagenomics of microbial entire communities (genomes derived from single-cell experiments, stable isotope probing experiments, “mini” metagenomes, long-read sequencing experiments, and cocultures were excluded). For each sequencing experiment, we downloaded the corresponding raw reads and, where appropriate, filtered out animal-associated reads by mapping to the host genome using bbduk (*qhdist *= 1). Sequencing experiments downloaded from the NCBI SRA database were subsampled to the average number of reads across all compiled experiments (∼36 million) using seqtk (*sample -s 7*) if the starting read pair count exceeded 100 million. We then removed Illumina adapters and other contaminants from the remaining reads and further quality trimmed them using Sickle. The filtered read set was then mapped against all genomes assembled (or coassembled) from it using bowtie2 (default parameters). For mappings with a nonzero number of read alignments, abundance of each genome was calculated by counting the number of stringently mapped reads (≥99% identity) using CoverM (*–min-read-percent-identity 0.99*) (https://github.com/wwood/CoverM) and dividing by the total number of reads in the quality-filtered read set. In most cases where genomes were derived from coassemblies of multiple sequencing experiments, we computed the abundance for each sample individually and then selected the one with the highest value as a “representative” sample for downstream analyses. To account for lower sequence representation of coassembled genomes in individual samples, we considered genomes present if at least 10% of their sequence length was covered by reads.

### Co-occurrence analyses.

Each representative sample was then probed for co-occurrence patterns of CPR and potential host lineages. To account for across-study differences in binning procedures, quality-filtered read sets were instead reassembled using MEGAHIT (*–min-contig-len 1000*) and analyzed using GraftM (108) with a ribosomal protein S3 (rpS3) gpackage custom built from GTDB (release 05-RS95) (109). Recovered rpS3 protein sequences in each sample were clustered to form “species groups” at 99% identity using USEARCH cluster_fast (*-sort length -id 0.99*). For all samples with >0 marker hits, we then performed three downstream analyses to examine patterns of co-occurrence for various taxa. First, we counted the number of unique species groups in each sample taxonomically annotated as Saccharibacteria (“*c*__*Saccharimonadia*”) and Actinobacteria (“*p*__*Actinobacteriota*”), dividing the former by the latter to compute a species “richness ratio” for each sample (where *p*__*Actinobacteriota* did not equal 0). Animal-associated samples without detectable rpS3 from Actinobacteria were secondarily profiled for ribosomal protein L6 from Actinobacteria using the same methodology as described above.

Second, to examine the co-occurrence of Saccharibacteria and Actinobacteria within a phylogenetic framework, we inferred maximum-likelihood trees for the set of rpS3 marker genes recovered across samples. Species group sequences were clustered across samples to further reduce redundancy using USEARCH (as described above) and were combined with rpS3 sequences drawn from a taxonomically balanced set of bacterial reference genomes (13) as an outgroup. Saccharibacteria and Actinobacteria sequence sets were then aligned, trimmed, and used to build trees as described above for the 16-ribosomal-protein tree, with the exception of using trimal (*-gt 0.1*) (110) instead of BMGE. Species groups that cooccurred in one or more metagenomic samples were then noted. If a given Saccharibacteria species group exclusively cooccurred with an Actinobacteria species group in at least one sample, or Actinobacteria species groups belonging to the same order level in all samples, those linkages were labeled. Finally, experimental cocultures of Saccharibacteria and Actinobacteria from previous studies were mapped onto the trees. To do this, we compiled a list of strain pairs and their corresponding genome assemblies (see Table S4 at https://doi.org/10.5281/zenodo.4560554) and then used GraftM to extract rpS3 sequences from corresponding genome assemblies downloaded from NCBI. We then matched these rpS3 sequences to their closest previously defined species group using blastp (*-evalue 1e−3 -max_target_seqs 10 -num_threads 16 -sorthits 3 -outfmt 6*), prioritizing hits with the highest bitscore and alignment length. Reference rpS3 sequences with no match at ≥99% identity and ≥95% coverage among the species groups were inserted separately into the tree. We then labeled all experimental pairs of species in the linkage diagram.

Third, we profiled a subset of 43 metagenomes containing Gracilibacteria and Absconditabacteria for overall community composition. For each sample, we extracted all contigs bearing rpS3 and mapped the corresponding quality-filtered read set to them using bowtie2. Mean coverage for each contig was then computed using CoverM (*contig –min-read-percent-identity 0.99*), and a minimum covered fraction of 0.10 was again employed. Relative coverage for each order level lineage (as predicted by GraftM) was computed by summing the mean coverage values for all rpS3-bearing contigs belonging to that lineage. Where species groups did not have order-level taxonomic predictions, the lowest available rank was used. Finally, relative coverage values were scaled by first dividing by the lowest relative coverage observed across samples and then taking the base-10 log. For the reanalysis of 22 cyanobacterial mat metagenomes (34), the same approach was taken, and coverage profiles for rpS3-bearing scaffolds were correlated using the pearsonr function in the scipy.stats package.

### Proteome size, content, and enrichment.

We subjected all predicted proteins from the genome set to a two-part, *de novo* protein clustering pipeline recently applied to CPR genomes, in which proteins are first clustered into “subfamilies” and highly similar/overlapping subfamilies are merged using an HMM-HMM comparison approach (–coverage 0.70) (12) (https://github.com/raphael-upmc/proteinClusteringPipeline). For each protein cluster, we recorded the most common KEGG annotation among its member sequences and the percentage of sequences bearing this annotation (e.g., 69% of sequences in fam00095 were matched with K00852).

We then performed three subsequent analyses to describe broad proteome features of included CPR bacteria. First, we computed proteome size across habitats, defined as the number of predicted open reading frames (ORFs) per genome when considering genomes at increasing thresholds of completeness in single-copy gene inventories (75%, 80%, 85%, etc.). Second, we examined similarity between proteomes by generating a presence/absence matrix of protein families with 5 or more member sequences. We then used this matrix to compute distance metrics between each genome based on protein content using the ecopy package in Python (method=‘jaccard’, transform=‘1’) and performing a principal-coordinate analysis (PCoA) using the skbio package. The first two axes of variation were retained for visualization alongside environmental and phylogenetic metadata. Finally, we used the clustermap function in seaborn (*metric=‘jaccard’*, *method=‘average’*) to hierarchically cluster the protein families based on their distribution patterns and plot these patterns across the genome set. For each protein family, we also computed the proportion of genomes encoding at least one member sequence that belonged to each of the three CPR lineages and each broad environmental category (Fig. 4, bottom panel) (see custom code linked under “Data and software availability”).

We next identified protein families that were differentially distributed among genomes from broad environmental categories. For each protein family, we divided the fraction of genomes from a given habitat (“in-group”) encoding the family by the same fraction for genomes from all other habitats (“out-group”). In cases where no “out-group” genome encoded a member protein, the protein family was simply noted as “exclusive” to the “in-group” habitat. In all cases, we calculated the Fisher exact statistic using the *fisher_exact* function in scipy.stats (*alternative=‘two-sided’*). To account for discrepancies in genome sampling among lineages, we determined ratios and corresponding statistical significance values separately for each lineage. All statistical comparisons for a given lineage were corrected for false-discovery rate (FDR) using the *multipletests* function in statsmodels.stats.multitest (*method=“fdr_bh”*). Finally, we selected families that were predicted to be enriched or depleted in particular habitats. We considered enriched families to be those with ratios ≥5 and depleted families to be those that were encoded in 10% or fewer of genomes from a given habitat but present in 50% or more of genomes outside that environmental category. Retaining only those comparisons with corrected Fisher’s statistics at FDR of ≤0.05 resulted in a set of 926 unique, differentially distributed protein families for downstream analysis.

### Analysis of putative rhodopsins.

Protein sequences from the CPR bacteria (fam11249) were combined with a set of reference protein sequences spanning type 1 bacterial/archaeal rhodopsin and heliorhodopsin (111). Sequences were then aligned using MAFFT (*–auto*), and a tree was inferred using IQ-TREE (-m TEST -st AA -bb 1000). Alignment columns with 95% or more gaps were trimmed manually in Geneious for the purposes of visualization. Transmembrane domains were identified by BLASTp searches (https://blast.ncbi.nlm.nih.gov/Blast.cgi), and conserved residues were defined by manual comparison with an annotated alignment of previously published reference sequences (112).

### Processes driving protein family evolution.

To examine the evolutionary processes shaping the differentially distributed protein families, we next subjected each family to an automated gene-species tree reconciliation workflow adapted from reference 92. Briefly, for each family, truncated sequences (defined as those with lengths less than 2 standard deviations from the family mean) were removed and the remaining sequences were aligned with MAFFT (*–retree 2*). Resulting alignments were then trimmed using trimal (*-gt 0.1*) and used to infer maximum-likelihood phylogenetic trees using IQ-TREE with 1,000 ultrafast bootstrap replicates (*-bnni -m TEST -st AA -bb 1000 -nt AUTO*). We removed reference sequences from the inferred species tree and rooted it on the branch separating Saccharibacteria from the monophyletic clade containing Gracilibacteria and Absconditabacteria. A random sample of 100 bootstrap replicates wasthen used to probabilistically reconcile each protein family with the pruned species tree using the ALE package (*ALE_undated*) (93). Estimates of missing gene fraction were derived from the CheckM genome completeness estimates described above. We then calculated the total number of originations (horizontal gene transfer from non-CPR bacteria, or *de novo* gene formation), within-CPR horizontal transfers, and losses over each nonterminal branch and mapped branch-wise counts for each event to a species-tree cladogram in iTOL (107).

### Data and software availability.

All accession information for the genomes analyzed in this study is listed in Table S1 at https://doi.org/10.5281/zenodo.4560554. Genomes as well as custom code for the described analyses are also available on GitHub, https://github.com/alexanderjaffe/cpr-crossenv. All supplemental figures, tables, and files are available through Zenodo (https://doi.org/10.5281/zenodo.4560554).
